# The Voluntary Hospitals and Parliament

**Published:** 1912-12-14

**Authors:** 


### THE VOLUNTARY HOSPITALS AND PARLIAMENT.

### Mr. Masterman Turns His Back upon Himself.

In the House of Commons on Tuesday Sir Hildred Carlile again questioned the Government as to the institutional treatment of insured persons who cannot be adequately treated in their own homes under the National Insurance Act. We give the verbatim report of Sir Hildred Carlile's question and Mr. Masterman's answer:—

Sir Hildred Carlile: To ask Mr. Chancellor of the Exchequer (1) whether any estimate has been made, and, if so, what, of the number of beds that will be required for the institutional treatment of patients who cannot be adequately treated in their own homes under the National Insurance Act; and whether in that case he will lay the figures before the House and, in addition, give any estimates which have been compiled showing the average cost of this branch of the work per thousand patients which may be thrown on the revenue of the local health committees; and (2) if how, under the National Insurance Act, major operations are to be provided for; whether the health committees will in all cases have power to incur whatever expense may be necessary in arranging for these; whether, in that case, the patient will have any freedom of choice as to the operating surgeon; and whether the local health committees will be bound to accept the advice of the particular medical man and have recourse to the services of the operator he recommends.

Mr. Masterman: Medical benefit under the Act, as has been frequently defined, includes that service which can ordinarily be given by general practitioners who come on to the panel. I know of no reason why the present treatment of the working people in hospitals and other similar institutions should be disturbed by the National Insurance Act. If as a result of experience the voluntary hospitals find that they require State grants for the treatment of insured persons, the whole question of State control would necessarily be raised; and at present this is not contemplated under the Act. The other parts of the two questions therefore do not arise.

An Impossible Position for the Government.

Mr. Masterman's declaration that he knows of no reason why the present treatment of the working people in hospitals and other similar institutions should be disturbed by the National Insurance Act is a remarkable one as it stands. Mr. Masterman must be quite aware that the National Insurance Act has compelled employers and working people individually to make compulsory payments every week for sick and medical benefit under the Insurance Act. The aggregate amount of those payments is infinitely larger than anything they have paid before to hospitals and, we shrewdly suspect, to sick clubs. But, apart from this altogether, the reasons why the present treatment of working people in hospitals must be disturbed by the National Insurance Act are that the Government have not come to arrangement with the members of the medical profession, that the Insurance Act has upset and grievously disturbed the benevolent contributors to hospitals, that the sum derived by the hospitals from this source may be materially lessened or altogether lost, and that an immediate consequence of the National Insurance Act must be largely to increase the number of persons who will seek in-patient treatment at the voluntary hospitals. Here are a few of many incontrovertible reasons why it is amazing that Mr. Masterman should have declared in the House of Commons that he knows no reason why the present treatment of the working people in hospitals should be disturbed by the National Insurance Act.

The question is too serious not to be treated soberly, thoughtfully, intelligently, and courageously. We hope that members of Parliament will continue to question the Government until the whole basis of hospital treatment is placed once and for all upon a definite and declared basis which can be understood by everybody. In last week's issue of The Hospital we showed that Mr. Masterman implied by two answers he gave in reply to Sir J. Lonsdale and Sir C. Kinloch-Cooke that the view of the Government was that the *pro rata* system could be adopted under the Act. To-day he turns completely round and declares that he knows no reason why the voluntary hospitals should not continue their present treatment of the working people as free patients, and in effect that nothing has happened under the National Insurance Act to disturb these relations. The truth is that either an arrangement must be come to with the voluntary hospitals to enable them to co-operate with the Government upon a business basis, or Parliament and the country must make up its mind that there will be grievous trouble all over the United Kingdom, and an amount of heretofore unknown miseries added to the troubles of the working people, to whom Mr. Masterman refers in his answer of the 10th instant.

